# Multimodal connectome biomarkers of cognitive and affective dysfunction in the common epilepsies

**DOI:** 10.1162/netn_a_00237

**Published:** 2022-06-01

**Authors:** Raul Rodriguez-Cruces, Jessica Royer, Sara Larivière, Dani S. Bassett, Lorenzo Caciagli, Boris C. Bernhardt

**Affiliations:** McConnell Brain Imaging Centre, Montreal Neurological Institute and Hospital, McGill University, Montreal, Quebec, Canada; Department of Bioengineering, University of Pennsylvania, Philadelphia, PA, USA; Department of Physics and Astronomy, University of Pennsylvania, Philadelphia, PA, USA; Department of Electrical and Systems Engineering, University of Pennsylvania, Philadelphia, PA, USA; Department of Neurology, University of Pennsylvania, Philadelphia, PA, USA; Department of Psychiatry, University of Pennsylvania, Philadelphia, PA, USA; Santa Fe Institute, Santa Fe, NM, USA; Department of Clinical and Experimental Epilepsy, UCL Queen Square Institute of Neurology, London, United Kingdom

**Keywords:** Epilepsy, Cognition, Affect, Dysfunction, Connectomics, Neuroimaging, Network neuroscience

## Abstract

Epilepsy is one of the most common chronic neurological conditions, traditionally defined as a disorder of recurrent seizures. Cognitive and affective dysfunction are increasingly recognized as core disease dimensions and can affect patient well-being, sometimes more than the seizures themselves. Connectome-based approaches hold immense promise for revealing mechanisms that contribute to dysfunction and to identify biomarkers. Our review discusses emerging multimodal neuroimaging and connectomics studies that highlight network substrates of cognitive/affective dysfunction in the common epilepsies. We first discuss work in drug-resistant epilepsy syndromes, that is, temporal lobe epilepsy, related to mesiotemporal sclerosis (TLE), and extratemporal epilepsy (ETE), related to malformations of cortical development. While these are traditionally conceptualized as ‘focal’ epilepsies, many patients present with broad structural and functional anomalies. Moreover, the extent of distributed changes contributes to difficulties in multiple cognitive domains as well as affective-behavioral challenges. We also review work in idiopathic generalized epilepsy (IGE), a subset of generalized epilepsy syndromes that involve subcortico-cortical circuits. Overall, neuroimaging and network neuroscience studies point to both shared and syndrome-specific connectome signatures of dysfunction across TLE, ETE, and IGE. Lastly, we point to current gaps in the literature and formulate recommendations for future research.

## Author Summary

Epilepsy is increasingly recognized as a network disorder characterized by recurrent seizures as well as broad-ranging cognitive difficulties and affective dysfunction. Our manuscript reviews recent literature highlighting brain network substrates of cognitive and affective dysfunction in common epilepsy syndromes, namely temporal lobe epilepsy secondary to mesiotemporal sclerosis, extratemporal epilepsy secondary to malformations of cortical development, and idiopathic generalized epilepsy syndromes arising from subcortico-cortical pathophysiology. We discuss prior work that has indicated both shared and distinct brain network signatures of cognitive and affective dysfunction across the epilepsy spectrum, improves our knowledge of structure-function links and interindividual heterogeneity, and ultimately aids screening and monitoring of therapeutic strategies.

## INTRODUCTION

Epilepsy is a neurological disorder characterized by recurrent seizures, affecting around 1% of the world population (approximately 50 million people) (GBD 2016 Neurology Collaborators, 2019). While seizures can be controlled with antiseizure medications in most patients, 30–40% of individuals are drug resistant and are at higher risk of widespread and cumulative brain damage (Bernasconi & Bernhardt, 2010; Bernhardt et al., 2009b, 2013b; Caciagli et al., 2017; Coan et al., 2009; Galovic et al., 2019). Moreover, cognitive impairment has been reported to encompass multiple domains (Loring et al., 2004), including memory, executive function, and language abilities. Difficulties in mood and emotion regulation are also increasingly recognized, challenging patient well-being and quality of life (Bell et al., 2011; Hermann et al., 2009). Collectively, these findings underscore the broad impact of epilepsy on brain health and function and emphasize the importance of shifting our understanding and clinical management of epilepsy as a disorder characterized by more than seizures (Selassie et al., 2014).

Temporal lobe epilepsy (TLE) related to mesiotemporal sclerosis as well as extratemporal epilepsy (ETE) related to malformations of cortical development are among the most common drug-resistant epilepsy syndromes. While both TLE and ETE have been traditionally labeled as ‘focal’ epilepsies related to a confined brain region, they are increasingly understood as system-level disorders of interconnected networks (Gleichgerrcht et al., 2015; Larivière et al., 2021; Tavakol et al., 2019). A growing body of neuroimaging as well as histopathological findings demonstrates diffuse network anomalies in both syndromes, affecting regions beyond the area responsible for the onset of seizures (Bernhardt et al., 2013a, 2015; Caciagli et al., 2014). Moreover, emerging work combining neuroimaging and cognitive testing suggests clear associations between atypical brain connectivity and measures of cognitive as well as affective function. Idiopathic generalized epilepsies (IGE), on the other hand, constitute approximately 20% of all patients with epilepsy and include childhood absence epilepsy, juvenile absence epilepsy, juvenile myoclonic epilepsy, and epilepsy with generalized tonic-clonic seizures alone. Neuroimaging studies in IGE have detected widespread anomalies in brain structure, connectivity, and function—paralleling mounting evidence in TLE and ETE on system-level dysfunction. Furthermore, multiple studies assessing cognitive abilities point to syndrome-specific impairments with, for example, measurable deficits in executive function as well as social and affective disturbances in patients with juvenile myoclonic epilepsy, and visuospatial and language deficits in absence epilepsies (Ratcliffe et al., 2020; Guida et al., 2019). Collectively, these studies suggest that widespread network anomalies contribute not only to the clinical manifestations of the disease, such as seizure burden and treatment response, but also to cognitive and affective difficulties affecting many patients’ lives.

**Figure 1.**
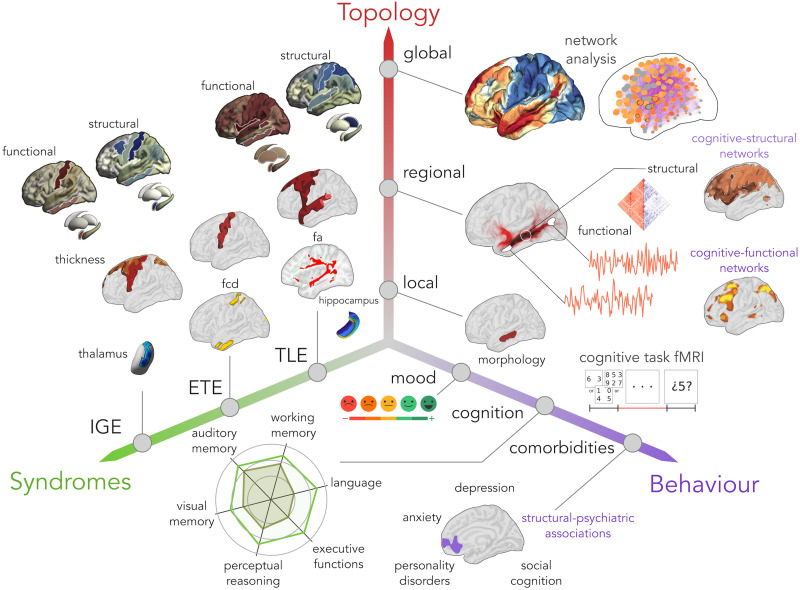
Investigating cognitive and affective dysfunction across the common epilepsies requires an integrative approach, combining multidimensional behavioral phenotyping, multimodal neuroimaging, and, ideally, the inclusion of a spectrum of epilepsy syndromes. In the current review, we summarize the literature that has so far populated this space in temporal lobe epilepsy (TLE), extratemporal epilepsy (ETE), and idiopathic generalized epilepsy (IGE).

Advances in multimodal neuroimaging and connectome analysis have contributed to our growing understanding of the structural and functional organization of brain networks (Larivière et al., 2018) and promise to provide candidate metrics that could inform clinical care (Larivière et al., 2021). In this targeted review, we will survey recent literature assessing connectome biomarkers of cognitive and affective dysfunction across common epilepsy syndromes, notably TLE, ETE, and IGE. Specifically, we will discuss the current literature on structural and functional network anomalies in each of these syndromes, outline the prevailing notions of syndrome-related cognitive dysfunction, and summarize emerging literature relating network neuroscience findings to neuropsychological assessments. Overall, findings support both shared and syndrome-specific patterns of structural and functional brain network reorganization across different spatial scales, motivating an integrative approach combining multidimensional behavioral phenotyping, multimodal neuroimaging, and, ideally, the inclusion of a spectrum of epilepsy syndromes (Figure 1). To conclude, we will highlight gaps in the literature and make recommendations for future research on brain and cognition in the common epilepsies.

## TEMPORAL LOBE EPILEPSY

The most common and widely studied drug-resistant epilepsy in adults is temporal lobe epilepsy (TLE). This syndrome is traditionally associated with variable degrees of histopathological alterations within mesial temporal lobe structures, such as the hippocampus, amygdala, and entorhinal cortex (Blumcke et al., 2013; Thom, 2014; Thom et al., 2009). Mounting histopathological and neuroimaging literature, however, challenges the notion that the condition can be reduced to localized pathology in the mesiotemporal lobes. Histological data suggests that mesiotemporal alterations often co-occur with broad rearrangements of cortical architecture, together with cell loss and gliosis in temporal and extratemporal cortices as well as subcortical structures, particularly the thalamus (Blanc et al., 2011; Margerison & Corsellis, 1966). These findings have been confirmed in vivo, based on structural MRI assessments applying volumetric analysis, as well as with automated surface-based measures (Bernhardt et al., 2008, 2012, 2015, 2016; Lin et al., 2007; McDonald et al., 2008). These studies have reported, in both single and multisite datasets, that TLE is often associated with diffuse cortical atrophy affecting mesiotemporal, lateral temporal, frontal, and centroparietal cortices, as well as profound atrophy of several subcortical structures (Larivière et al., 2020a; Whelan et al., 2018). While the distribution of gray matter atrophy appears relatively widespread and bilateral, analysis of cortical asymmetries has pointed more directly to marked changes in ipsilateral structures, particularly in temporo-limbic regions. These findings have been complemented by quantitative analysis of other MRI contrasts, such as FLAIR/T2w imaging (Adler et al., 2018) as well as quantitative T1 relaxometry (Bernhardt et al., 2018), suggesting that structural alterations are broad, yet most concentrated in paralimbic areas ipsilateral to the seizure focus. Similarly, several diffusion MRI studies have described anomalies in structural connectivity and subcortical white matter microstructure, strongly affecting temporo-limbic pathways and with more moderate effect sizes in extralimbic collateral and commissural pathways (Concha et al., 2005, 2009, 2012; Focke et al., 2008; Liu et al., 2016). Analysis of brain function and connectivity, as measured by task-based and resting-state fMRI, has shown atypical functional interactions between temporal and extratemporal areas in TLE patients relative to controls, with findings most marked findings in temporo-limbic and default mode networks, systems known to be anatomically connected to the mesiotemporal lobe (Bernhardt et al., 2016; Larivière et al., 2020b). Overall, despite sometimes diverging topographies of regional changes across different neuroimaging modalities, findings converge toward widespread network reorganization in TLE, likely modulated by anatomical connections to a mesiotemporal/paralimbic disease epicenter.

Considering cognitive function, a majority of TLE patients has traditionally been reported to show memory impairment, while around 30%–40% present with difficulties in language function (Reyes et al., 2018). Paralleling the widespread anomalies revealed by neuroimaging and histology, an increasing body of work suggests that impairments in executive (Lutz & Helmstaedter, 2005), sensory, and motor functions are also prevalent in TLE (Hermann et al., 2009; Lin et al., 2012; McAndrews & Cohn, 2012; Saling, 2009). As such, the landscape of cognitive difficulties in TLE emphasizes a broad profile of impairments across patients affecting multiple cognitive domains. Adding to the complexity of the cognitive dysfunction landscape in TLE, impairments are also quite variable across patients. Several groups have previously suggested the existence of graded abnormalities across TLE patient subgroups, with approximately a third of patients presenting with marked dysfunction while the remaining patients show mild or no measurable impairments compared to age-matched healthy individuals (Rodriguez-Cruces et al., 2018). In TLE, the degree and laterality of structural pathology in the hippocampus have been associated with verbal memory and language impairments, with patients with left sided and more marked pathology showing greater impairment (Dabbs et al., 2009; Stewart et al., 2009). Furthermore, functional connectivity measures of the hippocampus and cortical networks have been used to probe language lateralization (Benjamin et al., 2017; Berl et al., 2013; Lopes et al., 2019) and to predict deficits in episodic memory (Li et al., 2021). In the latter study, the investigators reported an atypical organization of functional connections that link the hippocampus to widespread cortical areas; the data suggests that the structural reorganization of the hippocampus and its functional interactions with the rest of the brain underpins reorganization of memory networks toward a potentially less efficient functional architecture (Li et al., 2021). Such findings are in line with earlier fMRI findings in TLE patients during memory tasks, showing a reorganization of both ipsilateral and contralateral hippocampal and extrahippocampal functional networks (Alessio et al., 2013; Bigras et al., 2013; Jokeit et al., 2001; Sidhu et al., 2013). Other work studied intrinsic functional hubs in TLE and reported an association between functional network reorganization and both language and memory impairment in patients (Roger et al., 2020). Together, these studies emphasize the role of (1) hippocampal-neocortical subnetworks and (2) structural alterations in the mesiotemporal lobes in determining cognitive deficits in TLE, particularly relating to memory and language function.

**Figure 2.**
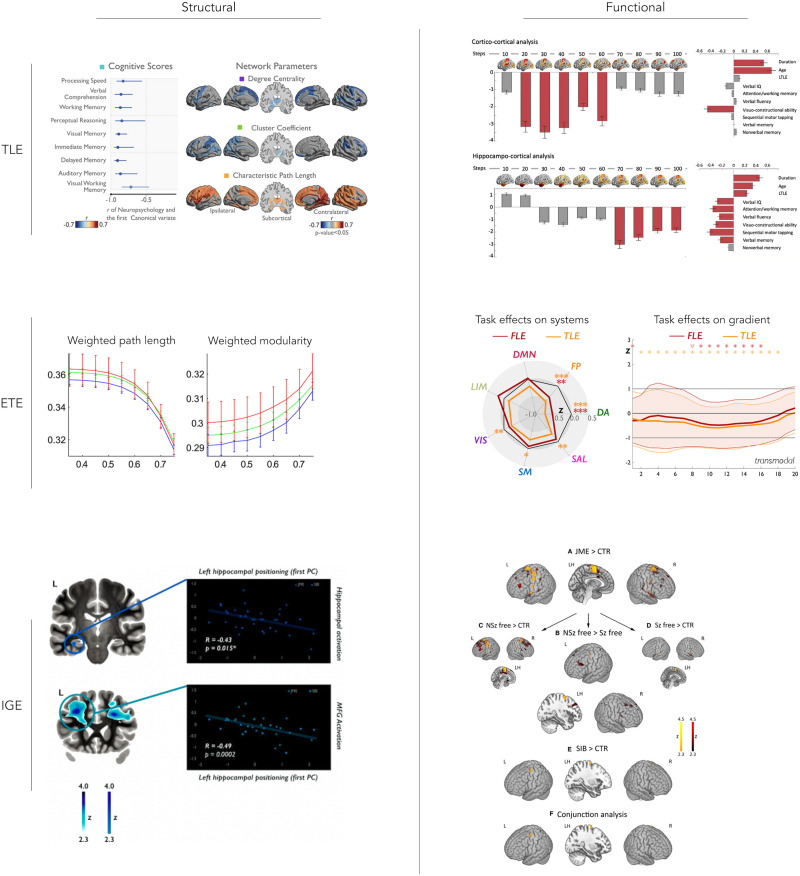
Prior multimodal imaging studies on connectome markers of dysfunction in the common epilepsies. In temporal lobe epilepsy (TLE; top row), structural network measures were related to cognitive dysfunction across multiple domains (top left) (Rodriguez-Cruces et al., 2019). Using stepwise functional connectivity analysis, alterations in hierarchical functional network organization were shown to reflect multidomain cognitive impairment (top right) (Fadaie et al., 2021). In extratemporal epilepsy (ETE; middle row), a study focusing on children with frontal lobe seizures demonstrated that despite the absence of significant differences in structural network parameters in patients, structural modularity increased with more marked cognitive impairment (middle left) (Vaessen et al., 2014) . When comparing fMRI activation patterns in a verbal working memory task, frontal lobe epilepsy patients showed targeted reductions in the recruitment of specific networks, notably fronto-parietal and dorsal attention systems, while effects in TLE patients were more widespread (middle right) (Caciagli et al., 2022). As for idiopathic generalized epilepsy syndromes (IGE; bottom row), a study in patients with juvenile myoclonic epilepsy and their unaffected siblings investigated left hippocampal shape/positional anomalies and found associations to atypical activation during a verbal memory task (bottom left) (Caciagli et al., 2019). Enhanced recruitment of motor systems during cognitive tasks, construed as an imaging phenotype in both patients and their unaffected siblings, was seen when assessing both memory and language tasks combined (bottom right) (Caciagli et al., 2020).

A separate and complementary series of studies identified associations between structural connectivity measures and cognitive phenotypes in TLE. There has been robust evidence for an association between diffusion abnormalities of deep white matter fiber tracts as well as the superficial white matter, and reduced language and memory performance in patients (Reyes et al., 2019). In one study, the authors combined dimensional and categorical multivariate approaches to show that more marked degrees of impairment across several cognitive functions related to overall less efficient and less interconnected white matter network organization (Figure 2, top left) (Rodriguez-Cruces et al., 2019). Another study leveraged communication models, which simulate functional interactions from structural connectivity data, and reported that overall delays in regional interactions related to broad impairments in several cognitive domains (Girardi-Schappo et al., 2021). Moving toward a biomarker evaluation framework, connectome-based machine learning—with cross-validation and the use of an independent hold-out dataset—has shown utility in predicting memory and language impairment, demonstrating outlook that brain network models can aid in indexing patient-specific functional impairments (Balachandra et al., 2020; Kaestner et al., 2020). In one of these studies, structural connectivity features achieved improved performance when they were combined with hippocampal imaging features, pointing to benefits of combining targeted assessments of the mesiotemporal epicenter with large-scale network models (Balachandra et al., 2020). Thus, the above studies emphasize the importance of efficient network communication in preserving cognition in TLE patients, and hold promise as robust biomarkers of cognitive difficulties in this population.

A growing body of studies, including combined phenotyping and neuroimaging efforts such as the Epilepsy Connectome Project, also assessed the utility of resting-state fMRI connectivity measures in the study of cognitive dysfunction in TLE. Combining resting-state fMRI connectivity analysis, morphological assessment, and supervised machine learning, a previous study assessed structural and functional brain aging in TLE patients, and reported associations between accelerated aging and cognitive decline in patients, particularly with regard to fluid abilities (Hwang et al., 2020). These findings are paralleled by reports of regional and global alterations in intrinsic functional network organization in TLE, showing associations between network alteration in the temporal lobe and clinical measures on the one hand, and global network clustering and cognitive decline on the other hand (Struck et al., 2021). Another recent study leveraged stepwise functional connectivity analysis, an approach tapping into putative cortical hierarchical organization, and showed associations between atypical sensory-fugal and hippocampo-cortical organization in TLE and cognitive dysfunction across multiple domains (Figure 2, top right) (Fadaie et al., 2021). Several studies have furthermore used resting-state fMRI to examine functional connectivity changes between brainstem nuclei of the ascending arousal system as well as cortical and subcortical regions (Englot et al., 2017, 2018). The authors reported overall reduced connectivity in patients, which reflected an increased frequency of seizures with impaired awareness and disease severity measures, alongside with impairments in verbal IQ, attention, executive function, language, and visuospatial memory (Englot et al., 2017, 2018). These findings in part recapitulate experimental work in animal models that have suggested an implication of subcortico-cortical loops in TLE pathophysiology and functional impairment. By using deep brain stimulation of specific thalamic divisions in animal models, it was demonstrated that cortical slow-wave activity can be reduced, which could also lead to reduced postictal behavioral impairment, supporting a potential therapeutic value of modulating subcortico-cortical loops (Xu et al., 2020).

Cognitive dysfunction may co-occur with socio-affective difficulties. A substantial proportion of patients suffer from depressive symptoms, anxiety, and personality disorders (Kanner et al., 2012; Tellez-Zenteno et al., 2007). As for cognitive dysfunction, affective symptoms may have a considerable impact on patient well-being and quality of life. In one previous study, the investigators related self-reports from the Beck Depression Inventory to structural and functional connectivity measures in both TLE patients and controls (Chen et al., 2012; Kemmotsu et al., 2013). The authors observed that structural and functional organization of fronto-limbic circuits, particularly connectivity between the hippocampus and anterior frontal regions, was associated with depressive symptoms. Fronto-limbic involvement was also suggested by one study relating resting-state fMRI connectivity measures to measures of neuroticism, depression, and anxiety (Doucet et al., 2013). The authors showed that neuroticism, a personality trait that is associated with depressive symptoms relates to atypical functional connectivity between mesiotemporal and frontal lobe regions, with connectivity alterations that were partially overlapping to those related to depression and anxiety symptoms (Rivera Bonet et al., 2020). In an earlier study, the authors also reported associations between affective symptoms and reductions in gray matter volumes, again targeting both frontal and temporal neocortices, as well as mesiotemporal lobe structures such as the hippocampus and amygdala (Rivera Bonet et al., 2019). Collectively, previous efforts to study socio-affective functioning in TLE highlight the role of fronto-limbic circuits, as well as the structural integrity of these regions, in mood-related symptoms.

## EXTRATEMPORAL LOBE EPILEPSY

Extratemporal lobe epilepsy (ETE) refers to a broad class of location-related syndromes in which the seizures originate from regions outside of the temporal lobe. The most common ETE subsyndrome is frontal lobe epilepsy, which accounts for approximately 50% of ETE patients (Delev et al., 2019; Lee et al., 2008). Although lesional etiologies are quite variable in ETE, many patients present with a suspected or histologically confirmed malformation of cortical development, such as focal cortical dysplasia (type-1 or type-2), heterotopia (subcortical nodular, band), or polymicrogyria. Some of these lesions may be subtle and overlooked upon conventional radiological examination, contributing to diagnostic uncertainty and difficulties in offering targeted surgical therapy (Bernasconi et al., 2011; Bernasconi & Bernasconi, 2011). While significant effort has been invested in characterizing malformations in single patients by using structural MRI and, increasingly, connectomics techniques (Bernasconi et al., 2001; Colliot et al., 2005; S. Hong et al., 2017a, 2017b; Wang et al., 2015), recent studies have also characterized the whole-brain substrates of ETE cohorts at a group level. These studies have shown that structural and functional network substrates across different ETE subsyndromes likely vary with respect to the primary lesional etiology. Patients with type-1 focal cortical dysplasia (a late-stage malformation associated with subtle alterations in cortical architecture and intensity) present with widespread cortical thinning relative to controls. In contrast, patients with type-2 dysplasia (early-stage cortical malformations that manifest on MRI as cortical thickening and interface blurring, together with FLAIR/T2w intensity changes; Hong et al., 2014, 2015) present with patches of increased cortical thickness beyond the primary lesional perimeter (Hong et al., 2016). A previous network analysis of frontal lobe epilepsy patients with different cortical malformations revealed a gradient of functional and structural network anomalies, showing only subtle structural network reorganization in type-2 dysplasia, moderate effects in heterotopias, and maximal changes in late-stage malformations such as polymicrogyria (Hong et al., 2017a). Findings were paralleled by graded functional network anomalies, pointing to reductions in functional network efficiency across the malformation spectrum, again being more pronounced in late-stage cortical malformations such as polymicrogyria than in early-stage proliferative etiologies such as type-2 dysplasia (Hong et al., 2017a). Previous work on ETE, thus, emphasizes the importance of considering the developmental etiology of lesional subtypes to understand structural and functional network anomalies in specific patient subgroups.

In ETE, the landscape of cognitive impairment is less well characterized than in TLE, owing to heterogeneity of the patients’ primary etiology and variable extent of the epileptogenic network. Findings nevertheless suggest a modulation of impairments with respect to the location of the disease epicenter. For instance, ETE patients with a suspected or confirmed anomaly in the frontal lobes will often present with executive dysfunction (Braakman et al., 2011; Exner et al., 2002; Patrikelis et al., 2016; Upton & Thompson, 1996; Verche et al., 2018), ranging from impaired attention to difficulties in goal-oriented behaviors. Verbal competencies also appear impaired (Helmstaedter et al., 1996; Risse, 2006), and reports have pointed to poorer motor coordination and reduced psychomotor speed (Upton & Thompson, 1996, 1997). Patients with lesions in central and posterior regions may show deficits in visuospatial functions, often together with attentional and motor dysfunction. Similar heterogeneity exists even within subgroups of patients with the same lesional subtype, where deficits can range from no apparent cognitive impairment to marked disability.

Studies assessing brain substrates of cognitive deficits in patients with ETE are less frequent than in TLE and are also more difficult to aggregate due to the above-mentioned heterogeneity across the patient spectrum. However, prior work in ETE samples (sometimes mixed with TLE) reported structural and functional connectivity alterations that relate to overall reductions in cognitive function (Vaessen et al., 2012, 2013). In mixed focal epilepsy cohorts including patients with ETE, prior work highlighted frontotemporal connectivity abnormalities and global alterations of functional network architecture during language tasks (Vlooswijk et al., 2010, 2011a), and prefrontal connectional rearrangements during verbal working memory (Vlooswijk et al., 2011b). In children with frontal lobe epilepsy, recent studies showed altered functional connectivity among fronto-temporo-parietal cortices during a working memory task compared to controls (Braakman et al., 2013), and also reported an association between atypical functional network modularity, consisting of reduced long-range connectivity and increased short-range connections, and reductions in cognitive performance (Vaessen et al., 2013). Notably, associations between cognitive impairment and network topology have been reported in structure as well as function. For example, structural networks estimated from diffusion MRI tractography evince marked alterations in segregation and integration in patients with more severe cognitive impairment (Figure 2, middle left) (Vaessen et al., 2012); similarly, increased clustering in structural covariance networks derived from T1-weighted data was not only associated with markers of disease severity but also with lower general intelligence (Drenthen et al., 2018). In a recent study, we combined a comprehensive cognitive profiling of language and working memory abilities with task-based fMRI analysis and connectome-level contextualization in a cohort with frontal lobe epilepsy, and compared findings to both patients with TLE and healthy controls (Figure 2, middle right) (Caciagli et al., 2022). Our study showed that working memory and language impairment in frontal lobe epilepsy was associated with reduced activation across attentional and executive systems, together with an attenuated deactivation of default mode regions, suggesting a reorganization of functional recruitment at the systems level. While atypical activation patterns were similar to those in TLE patients, reductions in default mode network deactivations appeared more marked in frontal lobe epilepsy patients, whereas those with TLE presented with less activations in posterior language areas during semantic tasks (Caciagli et al., 2022). Collectively, these findings provide emerging evidence of both shared and syndrome-specific impacts of different location-related epilepsies on brain functional networks. Moreover, this work broadly illustrates the utility of network neuroscience approaches in capturing important substrates that contribute to interpatient variability in cognitive dysfunction.

Studies of socio-affective functioning are less common than studies of cognitive function in ETE, but represent an important and growing area of research. One previous study in a cohort of 40 frontal lobe epilepsy cases reported that 25% have elevated anxiety scores, 40% have either elevated depression or elevated anxiety scores, and 25% have depression scores above the clinical cutoff (Tang et al., 2012). These values appear higher than in patients with generalized epilepsies (Tang et al., 2012), but are not as high as those observed in TLE (Helmstaedter, 2001). Furthermore, prior work highlights that executive dysfunction in frontal lobe epilepsy can co-occur with reduced response inhibition, hyperactivity, obsession, and addictive behaviors that may interfere with overall adaptation to everyday life (Helmstaedter, 2001). While there is abundant literature on behavioral and emotional difficulties in patients with specific lesions, including foundational case studies in patients with frontal lobe lesions, there are, to our knowledge, no systematic assessments of the relationship between whole-brain connectome architecture and affective difficulties in ETE patients.

## IDIOPATHIC/GENETIC GENERALIZED EPILEPSY

Idiopathic generalized epilepsy (IGE) refers to a group of epilepsy syndromes with likely polygenetic inheritance, and characterized by generalized spike and slow-wave discharges on EEG. While multiple syndromes are subsumed under the umbrella term of IGE (Andermann & Berkovic, 2001; International League Against Epilepsy, 1989; Nordli, 2005), converging evidence supports a key role of thalamo-cortical networks (Kwan et al., 2011). Fronto-thalamo-cortical involvement during generation and propagation of generalized seizures has been solidified by electrophysiological work (Blumenfeld, 2003; Gotman et al., 2005). Structural MRI studies complemented these findings, by indicating gray matter volume reductions in both the thalamus and neocortex (Bernhardt et al., 2009a), albeit to a lesser extent than in syndromes such as TLE (Weng et al., 2020; Whelan et al., 2018). In addition to atrophy, recent work also pointed to atypical cortical folding and cortex-cortical distance relationships in juvenile myoclonic epilepsy, with the latter also being present in nonaffected siblings (Wandschneider et al., 2019). Atypical cortical folding may signify perturbations in underlying brain connections; resting-state fMRI approaches have indeed supported atypical organization of cortico-thalamic networks in different IGE syndromes, showing aberrant connectivity when seeding from either cortical or thalamic regions (Wang et al., 2011, 2012, 2019). Similar reports have appeared in diffusion MRI studies, which showed atypical structural connectivity between thalamic and medial frontal regions in IGE subgroups with juvenile myoclonic epilepsy (JME) (O’Muircheartaigh et al., 2012).

The cognitive landscape of IGE is also characterized by atypical function across several domains. A systematic review and meta-analysis conducted across 26 studies revealed broad reductions in cognitive function across multiple domains in IGE patients, involving generalized cognitive ability, fluid/crystallized intelligence, processing speed, and memory abilities (Loughman et al., 2014). Impairments were observed in studies assessing mixed IGE cohorts, but also when considering specific IGE subsyndromes such as JME, childhood absence epilepsies, as well as epilepsy with generalized tonic-clonic seizures alone. Executive dysfunction represent a common trait across IGE subsyndromes (Ratcliffe et al., 2020), pointing to frontal lobe involvement, while impairment of visuospatial abilities may generally be subtle (Loughman et al., 2014). Another review focusing on childhood absence epilepsy also showed reductions in verbal skills across language and learning tasks, as well as mild impairments in executive functions (Verrotti et al., 2015). In JME, several studies also point to impaired social cognition (Guida et al., 2019; Ratcliffe et al., 2020), which may possibly relate to the consistent frontal lobe dysfunction seen in the disorder. Profiles of cognitive difficulties in IGE are thus seemingly broad, with specific subsyndromes possibly showing more circumscribed patterns of impairment.

Taken together, studies using cognitive and neuroimaging measures have supported an overall perturbation in the organization of prefrontal-central-thalamic connections. A positron emission tomography study reported prefrontal and subcortical glucose hypometabolism in patients with JME and associations with reduced working memory and mental flexibility (McDonald et al., 2006). These findings are complemented by a combined structural and functional MRI study in JME patients and their unaffected siblings, which reported hippocampal shape and positional anomalies as well as atypical functional activations, alongside with associations to impairments in verbal memory (Figure 2, bottom left) (Caciagli et al., 2019). Further task fMRI studies have reported altered motor activation across a battery of cognitive tasks (Figure 2, bottom right) (Caciagli et al., 2020; Vollmar et al., 2011) and atypical connectivity between prefrontal and motor networks during a working memory paradigm in both JME patients and their unaffected siblings, supporting that these imaging phenotypes may constitute heritable traits (Caciagli et al., 2020; Wandschneider et al., 2014). Atypical functional connectivity between prefrontal and thalamic regions was also observed during a verbal fluency task (O’Muircheartaigh et al., 2012) and was shown to relate to both reduced verbal fluency and decremented structural connectivity between these regions. Other structural and diffusion MRI studies in JME also support a network basis for impairment, by underscoring associations between gray and white matter alterations in both cortical and thalamic regions and cognitive impairments in mental flexibility, language, and memory function (Caciagli et al., 2019; Caeyenberghs et al., 2015; O’Muircheartaigh et al., 2011). Compared to the broad literature on network correlates of dysfunction in JME, association studies between imaging measures and cognitive variables are less frequent in absence epilepsy (Ratcliffe et al., 2020). One study reported atypical cortical morphology and folding in frontal and temporal cortices and indirectly supported a potential contribution to reductions in verbal and performance IQ measures (Tosun et al., 2011). During a sustained attention paradigm, a functional imaging study revealed an association between lower activation of the medial frontal cortex and impaired performance, which coexisted with reduction of fronto-insular resting-state connectivity (Killory et al., 2011). Collectively, although the amount of work associating cognitive phenotypes with brain network information is more limited for certain IGE subsyndromes, the literature overall suggests that atypical wiring and cross-talk of thalamic and cortical regions may contribute to the sometimes rather broad impairments seen in IGE patients.

## CONCLUSIONS AND CURRENT GAPS

Neuroimaging and connectomics approaches are beginning to reveal structural, functional, and network level substrates in TLE, ETE, and IGE. These studies support both shared and syndrome-specific patterns of cortico-subcortical reorganization across different epilepsy syndromes, with TLE being associated with marked and widespread network reorganization that is particularly extensive in the proximity of the mesiotemporal epicenter. While memory and language deficits are the most commonly reported cognitive difficulties, mounting evidence suggests that white matter microstructure and functional alterations also contribute to dysfunction in other cognitive domains, such as executive function. Fronto-limbic alterations in brain structure and function also contribute to commonly observed affective comorbidities, including depression and anxiety, and may potentially underpin other traits associated with TLE, such as neuroticism. In ETE, neuroimaging and connectomics studies suggest widespread impairments in structural and functional brain network organization, which seems to be modulated by both location and etiology of the primary lesion. For example, ETE patients with a suspected or confirmed anomaly in frontal circuits will often present with executive dysfunction as well as reduced verbal competence, sometimes together with poorer motor coordination and reduced psychomotor speed. Emerging literature has furthermore begun to reveal structural and functional substrates of cognitive impairments in ETE, suggesting reorganization in multiple brain subnetworks, often characterized by atypical activation/deactivation patterns of large-scale functional systems. Finally, although the different syndromes subsumed under the “IGE” umbrella term are relatively heterogenous, consistent structural and functional imaging findings point to thalamo-cortical structural and functional dysfunction. Across different IGE syndromes, most work has been performed in JME, pointing to similar, albeit somewhat more subtle cognitive impairment than in ETE patients with a frontal lobe seizure focus.

It is plausible that brain network measures may ultimately serve as powerful intermediary phenotypes to study effects of biological as well as environmental factors on cognitive systems in people with epilepsy, including medication effects, disease status, and baseline genetic factors. Notably, genetic influences are increasingly recognized to play a major role in shared and distinct connectomic and cognitive phenotypic associations across common epileptic syndromes (Busch et al., 2014; Zhu et al., 2020). To obtain a more thorough understanding of these likely complex interactions, future studies are recommended that combine multimodal imaging and connectomics with genetic testing as well as rigorous clinical phenotyping, in order to obtain a comprehensive picture of mechanisms leading to cognitive and affective dysfunction.

By offering a more comprehensive characterization of whole-brain alterations, incorporation of brain network fingerprinting could possibly aid in the calibration and monitoring of therapeutic efforts in patients with epilepsy. As this review has outlined, brain network measures have been repeatedly shown to reflect interindividual differences in cognitive impairment in different epilepsy syndromes (Balachandra et al., 2020; Hermann et al., 2020; Kaestner et al., 2020; Reyes et al., 2019; Rodriguez-Cruces et al., 2019). As such, these techniques could be used for the screening and subtyping of patients that may in turn become candidates for targeted rehabilitative therapies. Furthermore, and beyond their increasingly recognized utility in predicting seizure freedom postsurgery (Gleichgerrcht et al., 2020), brain network measures may potentially also help in the prognostication of post-operative outcomes in cognitive and affective domains. A recent study has, for example, shown an association between resting-state fMRI degree centrality measures of the language network and postoperative decline in naming in 20 patients undergoing left anterior temporal lobe resections (Audrain et al., 2018), and a promising area of future research will thus investigate the association between pre- and postoperative network measures as well as pre- and postoperative cognitive and affective markers.

Epilepsy is truly more than a seizure condition, and it is thus important to continue to identify underlying mechanisms of cognitive function across the spectrum of common epilepsies. This expansion will involve increasing research efforts into less prevalent syndromes and further studying sources of interpatient variability. For these studies, we emphasize the need to combine multidimensional cognitive phenotyping approaches with multimodal neuroimaging, given the likely complementary power of structural and functional imaging techniques. As it is becoming increasingly evident from the current literature that cognitive and affective dysfunction in the epilepsies likely relates to atypical neural organization at different spatial and temporal scales, there will likely be an increased demand for advanced analysis approaches. Such analyses include multivariate associative techniques, multilayer network analysis, as well as machine learning approaches, and promise to address the likely complex associations between brain network organization and dysfunction, while also modeling effects of genetic, environmental, and disease factors. In TLE, prior clustering and multivariate associative studies are already beginning to shed light on covariations in atypical connectome organization and cognition, and further help to clarify sources of interindividual variations across the patient spectrum. Paralleling current trends in the study of the brain and mental health, we recommend cross-syndrome investigations that can identify shared and syndrome-specific effects on brain network organization and cognition. Such approaches will provide higher granularity in studying the panorama of cognitive and affective impairments and associated connectome anomalies in TLE, ETE, and IGE. Finally, to generalize from potential idiosyncrasies of specific epilepsy centers and to allow for an unbiased evaluation of network biomarkers in the prediction of dysfunction at the single-patient level, we recommend multisite data aggregation and analysis efforts such as ENIGMA-Epilepsy (Sisodiya et al., 2020), along with strategies for prospective as well as retrospective data harmonization at the level of brain and cognition alike.

## CITATION DIVERSITY STATEMENT

Recent work in several fields of science has identified a bias in citation practices such that papers from women and other minority scholars are undercited relative to the number of such papers in the field (Dworkin et al., 2020; https://github.com/dalejn/cleanBib). Here we sought to proactively consider choosing references that reflect the diversity of the field in thought, form of contribution, gender, race, ethnicity, and other factors. First, we obtained the predicted gender of the first and last author of each reference by using databases that store the probability of a first name being carried by a woman (Dworkin et al., 2020). By this measure (and excluding self-citations to the first and last authors of our current paper), our references contain 18.29% woman(first)/woman(last), 9.4% man/woman, 23.16% woman/man, and 49.15% man/man. This method is limited in that (1) names, pronouns, and social media profiles used to construct the databases may not, in every case, be indicative of gender identity and (2) it cannot account for intersex, nonbinary, or transgender people. Second, we obtained predicted racial/ethnic category of the first and last author of each reference by databases that store the probability of a first and last name being carried by an author of color (Sood & Laohaprapanon, 2018). By this measure (and excluding self-citations), our references contain 13.13% author of color (first)/author of color (last), 11.24% white author/author of color, 25.8% author of color/white author, and 49.84% white author/white author. This method is limited in that (1) names and Florida voter data to make the predictions may not be indicative of racial/ethnic identity and (2) it cannot account for Indigenous and mixed-race authors, or those who may face differential biases due to the ambiguous racialization or ethnicization of their names. We look forward to future work that could help us to better understand how to support equitable practices in science.

## AUTHOR CONTRIBUTIONS

Raul Rodriguez-Cruces: Conceptualization; Investigation; Visualization; Writing – original draft; Writing – review & editing. Jessica Royer: Investigation; Visualization; Writing – original draft; Writing – review & editing. Sara Larivière: Writing – review & editing. Dani S. Bassett: Writing – review & editing. Lorenzo Caciagli: Conceptualization; Writing – original draft; Writing – review & editing. Boris C. Bernhardt: Conceptualization; Funding acquisition; Investigation; Writing – original draft; Writing – review & editing.

## FUNDING INFORMATION

Raul Rodriguez-Cruces, Fonds de la Recherche du Québec – Santé, Award ID: FRQ-S 291486. Jessica Royer, Canadian Institute of Health Research, CIHR Fellowship. Boris C. Bernhardt, National Science and Engineering Research Council of Canada, Award ID: NSERC Discovery-1304413; Canadian Institute of Health Research, Award ID: FDN-154298; Canadian Institute of Health Research, Award ID: PJT-174995; SickKids Foundation, Award ID: NI17-039; Azrieli Center for Autism Research (ACAR-TACC), New Investigator Research Grant; BrainCanada, Future Leaders Research Grant; Helmholtz International Bigbrain Analytics and Learning Laboratory (Hiball), FRQ-S, and the Tier-2 Canada Research Chairs program, Tier-2. Sara Larivière, Canadian Institute of Health Research, CIHR doctoral award. Lorenzo Caciagli and Dani S. Bassett, NINDS (R01-NS099348). Dani S. Bassett, John D. and Catherine T. MacArthur Foundation; Alfred P. Sloan Foundation; the Paul Allen Family Foundation; and the ISI Foundation.

## TECHNICAL TERMS

Epilepsy: A common chronic condition characterized by recurrent seizures.
TLE: Temporal lobe epilepsy, one of the most common epilepsy syndromes and one that is often drug-resistant.
Mesiotemporal sclerosis: A lesion characterized by cell loss and/or gliosis of the mesiotemporal lobe structures, such as the hippocampus and entorhinal cortex, common in patients with TLE.
ETE: Extratemporal lobe epilepsy, a heterogenous group of epilepsy syndromes characterized by seizures originating from outside the temporal lobes.
Malformations of cortical development: A spectrum of lesions associated with atypical cortical development and a frequent cause of drug-resistant seizures.
IGE: Idiopathic generalized epilepsy, a group of epilepsy syndromes associated to generalized seizures that are believed to have a strong polygenetic basis.
Connectome: A systematic representation of the brain’s network (either structural or functional).
Biomarker: An objectively measured indicator of a biological process, normal or pathological, with potential diagnostic or prognostic utility for a given person/patient.
MRI: Magnetic resonance imaging.
JME: Juvenile myoclonic epilepsy, a common generalized epilepsy syndrome.
